# Impulse Oscillometry Findings in Children and Adolescents with Adrenal Insufficiency: An Exploratory Cross-Sectional Study

**DOI:** 10.3390/life16101764

**Published:** 2026-10-21

**Authors:** Ercan Yılmaz, Erdem Topal, İsmail Dündar, Zeynep Yamancan Yılmaz, Nezihe Koker Ozer, Emine Çamtosun

**Affiliations:** 1 Department of Pediatric Allergy and Immunology, Faculty of Medicine, Inonu University, 44280 Malatya, Turkey; yilmaz.ercan@inonu.edu.tr (E.Y.);; 2 Department of Pediatric Endocrinology, Faculty of Medicine, Inonu University, 44280 Malatya, Turkey; 3 Department of Pediatrics, Faculty of Medicine, Inonu University, 44280 Malatya, Turkey

**Keywords:** adrenal insufficiency, children, congenital adrenal hyperplasia, impulse oscillometry, peripheral airway function

## Abstract

Background: Airway function in children with adrenal insufficiency (AI) has not been well characterized. We evaluated impulse oscillometry (IOS) findings in pediatric AI and compared patients with congenital adrenal hyperplasia (CAH) and AI due to causes other than CAH (non-CAH AI). Methods: In this cross-sectional study, 52 children with AI underwent IOS. Oscillometric abnormalities were defined using z-score-based upper and lower limits of normal. IOS z-scores and clinical characteristics were compared between the CAH (n = 24) and non-CAH AI (n = 28) groups. Results: The median age was 16 years (IQR, 6 years; min–max, 4–18 years), and 53.8% of participants were male. CAH was the most common etiology (46.1%). Median (IQR) IOS values were 0.49 (IQR, 0.32) kPa·L^−1^·s for R5, 0.29 (IQR, 0.13) kPa·L^−1^·s for R20, 0.20 (IQR, 0.16) kPa·L^−1^·s for R5–R20, −0.13 (IQR, 0.11) kPa·L^−1^·s for X5, −0.01 (IQR, 0.08) kPa·L^−1^·s for X20, 1.74 (IQR, 1.30) kPa·L^−1^ for AX, and 20.8 (IQR, 5.32) Hz for resonance frequency (Fres). The most frequent abnormality was reduced X5 (21.2%), followed by elevated R5 (19.2%), elevated R5–R20 (19.2%), reduced X20 (15.4%), elevated AX (15.4%), elevated R20 (13.5%), and elevated Fres (7.7%). No significant differences were observed between the CAH and non-CAH AI groups in demographic characteristics, atopic diseases, or any IOS parameter (all *p* > 0.05). Conclusions: A subset of children with AI had IOS measurements outside published parameter-specific reference limits. Among individual IOS parameters, measurements outside the applied reference limits were most frequently observed for X5, R5, and R5–R20. Because no healthy control group was included and the reference equations were derived from populations different from the study population, these findings should be interpreted as exploratory.

## 1. Introduction

Adrenal insufficiency (AI) is a chronic endocrine disorder characterized by insufficient glucocorticoid production; although rare in childhood, it can be life-threatening. This clinical condition, which encompasses diseases with different etiologies such as primary adrenal insufficiency, secondary AI, and congenital adrenal hyperplasia (CAH), requires lifelong glucocorticoid replacement therapy. Thanks to advances in diagnostic methods and the standardization of replacement therapy in recent years, survival rates among children with AI have increased significantly; consequently, long-term complications have come to be regarded as increasingly important. However, the effects of chronic cortisol deficiency on the respiratory system have not yet been fully elucidated [1,2,3].

Glucocorticoids are hormones that play a critical role in fetal lung development. Whilst cortisol stimulates the maturation of type II alveolar cells, surfactant synthesis, and the development of the distal airways, it also plays a role after birth in regulating airway epithelial integrity, controlling inflammation, bronchial smooth muscle function, and extracellular matrix remodeling. Experimental studies have shown that glucocorticoid deficiency can enhance the inflammatory response, impair epithelial repair, and alter airway mechanics. Nevertheless, it remains largely unknown how these pathophysiological changes manifest in clinical airway function in children with AI [4,5,6].

The vast majority of existing studies have focused on adrenal crisis, infections, or the metabolic consequences of replacement therapy. Assessments of the respiratory system, however, are mostly limited to conventional spirometry. Conventional spirometry may be relatively insensitive to early functional changes in the peripheral airways, particularly when standard indices such as FEV_1_ remain within the normal range. Consequently, clinically silent peripheral airway dysfunction in children with AI may be overlooked [7,8].

In recent years, it has been shown that the peripheral airways are affected in the early stages of many chronic diseases. In conditions such as asthma, obesity, bronchopulmonary dysplasia, cystic fibrosis, and allergic rhinitis, peripheral airway involvement may develop before clinical symptoms appear and may be associated with the prognosis of the disease. Small airway dysfunction may be present before abnormalities become apparent in conventional spirometric parameters such as FEV_1_. Therefore, complementary methods for assessing peripheral airway function may provide additional information [9,10,11,12].

Impulse oscillometry (IOS) measures respiratory system impedance during quiet tidal breathing and requires minimal active cooperation, making it particularly useful in children. IOS parameters, including R5, R20, R5–R20, X5, AX, and resonance frequency (Fres), provide information on respiratory system resistance and reactance. These measurements reflect the combined mechanical properties of the airways, lung tissue, and chest wall and should not be interpreted as direct, anatomically specific measures of peripheral airway dysfunction. The European Respiratory Society (ERS) technical standards provide recommendations for measurement procedures, quality control, and reporting of respiratory oscillometry [9].

There are various biological mechanisms that may explain the impairment of peripheral airway function in adrenal insufficiency. Chronic cortisol deficiency may lead to low-grade systemic inflammation, impaired airway epithelial integrity, altered immune responses, and remodeling of the extracellular matrix. Furthermore, long-term glucocorticoid use in chronic adrenal insufficiency, hyperandrogenism, and metabolic changes may also exert additional effects on the respiratory system. Conversely, in secondary adrenal insufficiency, accompanying pituitary hormone deficiencies may affect lung development through different mechanisms. However, clinical data supporting these hypotheses are quite limited, and studies using IOS are virtually non-existent [3,7,13].

Given the paucity of clinical data regarding respiratory function in children with adrenal insufficiency, IOS may provide an exploratory means of characterizing airway mechanics in this population. However, the biological mechanisms outlined above cannot be tested by the current study design. All participants were receiving hydrocortisone replacement, and information on disease duration, cumulative glucocorticoid exposure, and hormonal control was unavailable. Therefore, this cross-sectional study cannot distinguish the potential contributions of cortisol deficiency, the underlying disease, glucocorticoid replacement, or other comorbid and metabolic factors to the observed IOS findings. Comparisons between the CAH and non-CAH AI groups are descriptive and exploratory and cannot establish the mechanisms underlying any observed differences. Any IOS abnormalities should also be interpreted cautiously, as their clinical significance requires correlation with respiratory symptoms, conventional pulmonary function tests, and longitudinal outcomes.

To the best of our knowledge, there is no comprehensive study that has assessed airway function using impulse oscillometry in a heterogeneous group of pediatric patients with adrenal insufficiency, described the frequency of IOS values outside published reference limits, and compared airway mechanics between children with CAH and those with non-CAH causes of adrenal insufficiency. Therefore, the aim of this study was to explore airway mechanics in children with AI using impulse oscillometry, to describe the frequency of abnormal IOS findings relative to reference values, and to compare oscillometric characteristics between children with CAH and those with other causes of adrenal insufficiency.

## 2. Materials and Methods

### 2.1. Study Design and Patient Population

This single-center, cross-sectional observational study assessed respiratory mechanics using IOS in children and adolescents receiving follow-up care for AI. This cross-sectional observational study is reported in accordance with the Strengthening the Reporting of Observational Studies in Epidemiology (STROBE) checklist. The study was conducted in collaboration between the Division of Pediatric Endocrinology and the Division of Pediatric Immunology and Allergic Diseases at the Faculty of Medicine, Inonu University. The study included children diagnosed with congenital adrenal hyperplasia, primary adrenal insufficiency, panhypopituitarism, adrenoleukodystrophy, craniopharyngioma, autoimmune adrenal insufficiency, Triple A syndrome, and AI caused by a melanocortin-2 receptor (MC2R) mutation. Immediately after obtaining ethics committee approval, potentially eligible patients were identified from the pediatric endocrinology outpatient clinic database and contacted by telephone. Families willing to participate were invited to the clinic for evaluation. During the study visit, demographic and clinical data were recorded using standardized case report forms, and all respiratory assessments were completed on the same day under the supervision of the study investigators. Care was taken to ensure that all participants were clinically stable at the time of IOS testing. Individuals experiencing an acute adrenal crisis, as well as children with established chronic pulmonary diseases expected to independently and persistently affect respiratory mechanics, congenital heart disease, neuromuscular disorders, prematurity, or a respiratory tract infection within the preceding four weeks, were excluded.

Asthma was defined as physician-diagnosed asthma documented in the medical records and was recorded as an atopic comorbidity rather than an exclusion criterion. However, children with a recent asthma exacerbation were not assessed. Of the five participants with asthma, two were receiving inhaled corticosteroid therapy, and three were not receiving asthma treatment. All five had controlled asthma at the time of IOS assessment. A sensitivity analysis was performed excluding these five participants, and parameter-specific frequencies of measurements outside the reference limits were recalculated.

### 2.2. Assessment of Clinical, Laboratory, and Treatment Characteristics

Patients’ age, sex, height, weight, and body mass index (BMI) were recorded. Patients’ height and weight measurements were taken using standard methods, and height z-scores and weight z-scores were calculated according to age and sex. Anthropometric assessments were performed in accordance with national reference data [14]. Information on AI etiology, previous adrenal crises, parental consanguinity, personal history of asthma, allergic rhinitis, or atopic eczema, and family history of atopy was obtained from the medical records.

With regard to treatment characteristics, the hydrocortisone dose (mg/m^2^/day) was recorded, along with the use of fludrocortisone, levothyroxine, growth hormone, desmopressin, antihypertensive drugs, antiandrogen therapy, oral contraceptives, testosterone, and aromatase inhibitors.

Laboratory data for all patients were obtained retrospectively from patient records. Eosinophil counts were recorded from peripheral complete blood counts, and total serum immunoglobulin E (IgE) levels were assessed based on the available data. The treatment agents used by patients with CAH were examined in detail. Whilst all patients were receiving hydrocortisone treatment, patients also receiving fludrocortisone treatment, antihypertensive treatment, antiandrogen treatment, oral contraceptive/estrogen treatment, aromatase inhibitor therapy, testosterone treatment, desmopressin treatment, Genotropin treatment, and levothyroxine treatment were recorded.

### 2.3. Impulse Oscillometry Measurement and Quality Control

Airway resistance and reactance were assessed using an impulse oscillometry system (Vyntus™ IOS, Vyaire Medical GmbH, Höchberg, Germany). Before each testing session, calibration of the IOS device was performed according to the manufacturer’s recommendations using a standard 3-L syringe and reference resistance.

IOS testing was conducted according to the technical recommendations for respiratory oscillometry issued by the European Respiratory Society (ERS) [9]. Participants were instructed to avoid heavy meals and vigorous exercise before testing. In participants using short-acting bronchodilators, these medications were withheld for at least 4–6 h prior to IOS assessment in accordance with the testing protocol. All children were clinically stable on the day of testing, and those with a recent respiratory tract infection were assessed only after meeting the predefined eligibility criteria. IOS testing was carried out in a quiet setting while participants were seated with the head in a neutral position and a nose clip in place. During the measurements, participants supported their cheeks and the floor of the mouth with their hands to reduce upper airway artifacts.

Quality-control criteria were specified before IOS testing and data analysis. For each recording, participants breathed quietly through the device mouthpiece for approximately 30–45 s while maintaining an adequate seal. A minimum of three technically acceptable recordings was required for each participant. Recordings were accepted only when tidal breathing remained stable and no technical artifacts, including coughing, swallowing, vocalization, air leakage, or irregular breathing, were present. Reproducibility was assessed using the coefficient of variation (CV) for R5, with a CV <15% required for inclusion in the analysis; the ERS technical standard recommends ≤15% in children [9]. Additional coherence thresholds of ≥0.80 at 5 Hz and ≥0.90 at frequencies ≥10 Hz were applied. All technically acceptable recordings were averaged to obtain the final IOS values, including when more than three acceptable recordings were available.

### 2.4. Reference Equations and Definition of Abnormal IOS Parameters

Airway resistance was characterized using R5, representing total respiratory resistance at 5 Hz; R20, representing resistance at 20 Hz and predominantly reflecting the more central airways; and R5–R20, representing frequency dependence of resistance and used as an index of peripheral airway involvement. Reactance was assessed using X5 and X20, together with the area of reactance (AX) and Fres. X5 was considered the principal low-frequency reactance parameter relevant to peripheral airway mechanics, whereas X20 was retained as a complementary descriptive measure of higher-frequency reactance. AX and Fres were additionally evaluated as integrated indices of respiratory system reactance.

Reference equations were selected according to participant age and IOS parameter using the reference sets incorporated into the SentrySuite software [version 3.20 (Vyaire Medical GmbH, Höchberg, Germany)]. Predicted values and z-scores for R5, R20, X5, X20, and Fres were generated by the device software using age-specific reference equations implemented in the system: Dencker equations for participants aged 2–11 years [15], Lechtenbörger equations for those aged 12–17 years, as described by Larsen et al. [16], and Vogel and Smidt equations for those aged 18–69 years [17]. Each participant’s IOS measurements were interpreted using the corresponding age-specific reference set implemented in the software. Because z-scores for R5–R20 and AX were not available from the age-specific reference modules in our SentrySuite configuration, these parameters were evaluated separately using the reference equations published by Gochicoa-Rangel et al. [18]. These equations were derived from 830 healthy nonsmoking participants aged 2.7–90 years, predominantly residing in Mexico City, Mexico. The reference models used multivariable segmented regression incorporating sex, age, height, and body mass index. For R5–R20 and AX, predicted values and z-scores were calculated using the publicly accessible IOS Z-score Calculator (https://ioszscore.manus.space/ accessed on 4 October 2026). The required demographic and anthropometric variables and the observed IOS values were entered for each participant. Supplementary Materials S1 provides an illustrative example of the calculator inputs and outputs, including predicted values, lower and upper limits of normal, and z-scores. Although the illustrative report displays all seven IOS parameters, only the calculator-derived results for R5–R20 and AX were used in the study; values for the remaining parameters were obtained from the age-specific reference modules in SentrySuite. Across all reference sets, abnormal values were defined using consistent z-score thresholds: z > +1.645 for R5, R20, R5–R20, AX, and Fres, and z < −1.645 for X5 and X20. The number and percentage of participants with abnormal values were calculated separately for each IOS parameter.

### 2.5. Statistical Analysis

Data analysis was performed using IBM SPSS Statistics for Windows (24.0, IBM Corp., Armonk, NY, USA). The distribution of continuous variables was assessed using the Shapiro–Wilk test. Variables showing a normal distribution are presented as mean ± standard deviation, whilst those not following a normal distribution are presented as the median (interquartile range (IQR)). Categorical variables were expressed as frequencies and percentages. Non-normally distributed continuous variables were compared between two independent groups using the Mann–Whitney U test. Exploratory Pearson correlation analyses were performed to assess associations of hydrocortisone dose with age, height, and absolute IOS parameters (X5, R5–R20, and AX), using available paired observations without adjustment for potential confounders. Categorical variables were compared using Pearson’s chi-square test or Fisher’s exact test. All tests were two-sided, with a nominal significance threshold of *p* < 0.05. Given the exploratory nature of the analyses, no adjustment for multiple comparisons was applied; therefore, *p*-values should be interpreted cautiously rather than as confirmatory evidence. A sensitivity analysis was performed by excluding the five participants with asthma (n = 47). The frequencies of IOS measurements outside the corresponding reference limits were recalculated, and IOS z-scores were compared again between the CAH and non-CAH AI groups using the same statistical approach.

## 3. Results

### 3.1. Baseline Demographic, Clinical, Laboratory, and Treatment Characteristics of the Study Population

A total of 52 children diagnosed with AI were included in the study. Twenty-eight of the patients (53.8%) were male. The median age was 16 years (IQR, 6 years; min–max, 4–18 years). Of the 52 participants, 3 (5.8%) were aged <6 years, 15 (28.8%) were aged 6–12 years, and 34 (65.4%) were aged 13–18 years. The mean z-scores for height, weight, and body mass index were −0.53 ± 1.59, −0.58 ± 2.05, and 0.66 ± 1.61, respectively. The most common cause of AI was congenital adrenal hyperplasia, observed in 24 patients (46.1%). This was followed by adrenoleukodystrophy and craniopharyngioma, each in 6 patients (11.5%); panhypopituitarism in 5 patients (9.6%); primary AI in 4 patients (7.7%); and MC2R mutation in 3 (5.7%) patients, followed by autoimmune AI and Triple A syndrome, each in 2 patients (3.8%). Four patients (7.7%) had a history of adrenal crisis, whilst 24 patients (46.2%) had a history of consanguineous marriage. When assessing atopic conditions, a history of asthma was identified in 5 patients (9.6%), allergic rhinitis in 5 patients (9.6%), and eczema in 6 patients (11.5%). Fifteen patients (28.8%) had a family history of atopy. The mean eosinophil count was 169.2 ± 135.2 cells/µL, whilst the mean total IgE level was 111.8 ± 74.2 IU/mL. Whilst all patients were receiving hydrocortisone treatment, 12 patients (23.1%) were taking fludrocortisone; 11 patients (21.2%) were taking levothyroxine; 7 patients (13.5%) were on antihypertensive treatment; 6 patients each (11.5%) were on antiandrogens, oral contraceptives/estrogen, and desmopressin; 5 patients (9.6%) were on growth hormone (Genotropin); and 2 patients each (3.8%) were on aromatase inhibitor and testosterone therapy. The mean hydrocortisone dose was calculated as 11.51 ± 5 mg/m^2^/day (Table 1).

### 3.2. Oscillometric Findings in Children with Adrenal Insufficiency

In children with adrenal insufficiency, the median (IQR) R5 value in impulse oscillometry assessment was 0.49 (IQR, 0.32) kPa·L^−1^·s, the R20 value was 0.29 (IQR, 0.13) kPa·L^−1^·s, the R5–R20 value was 0.20 (IQR, 0.16) kPa· L^−1^·s, the X5 value was −0.13 (IQR, 0.11) kPa·L^−1^·s, the X20 value was −0.01 (IQR, 0.08) kPa·L^−1^·s, the AX value was 1.74 (IQR, 1.30) kPa·L^−1^, and the Fres value was 20.8 (IQR, 5.32) Hz. When assessed according to z-score-based ULN/LLN criteria, the most common abnormal finding was a low X5 value in 11 patients (21.2%). This was followed by high R5 and high R5–R20 values in 10 patients each (19.2%), low X20 and high AX values in 8 patients each (15.4%), a high R20 value in 7 patients (13.5%), and a high Fres value in 4 patients (7.7%). Overall, 18 (34.6%) participants had at least one IOS parameter outside the applied reference limits, 8 (15.4%) had at least two, and 7 (13.4%) had at least three. These categories are cumulative, and individual parameter abnormalities may occur in the same participant. These percentages describe the frequency of measurements outside the selected parameter-specific reference limits within the study cohort and do not establish an increased frequency of respiratory dysfunction compared with healthy children (Table 2).

In exploratory Pearson correlation analyses, hydrocortisone dose showed a weak positive correlation with age (r = 0.282, *p* = 0.045). No statistically significant correlations were detected with height (r = 0.201, *p* = 0.157), absolute X5 values (r = 0.117, *p* = 0.412), absolute R5–R20 values (r = −0.160, *p* = 0.263), or absolute AX values (r = 0.008, *p* = 0.958; n = 51). These analyses were unadjusted for potential confounders, and all *p*-values were nominal and unadjusted for multiple comparisons.

### 3.3. Comparison of Demographic, Clinical, and Oscillometric Characteristics Between Congenital Adrenal Hyperplasia and Non-CAH Adrenal Insufficiency

Children with congenital adrenal hyperplasia (CAH) and those with non-CAH AI were compared in terms of demographic characteristics, the prevalence of atopic diseases, and IOS z-scores. The proportion of males was 37.5% (9/24) in the CAH group and 67.9% (19/28) in the non-CAH AI group (*p* = 0.056). Although this difference did not meet the prespecified threshold for statistical significance, the imbalance in sex distribution warrants consideration when interpreting the unadjusted between-group comparisons.

The median age was 14.5 (IQR, 6) years in the CAH group and 16 (IQR, 6) years in the non-CAH AI group (*p* = 0.570). No statistically significant between-group differences were observed in the frequencies of asthma [4 (16.7%) vs. 1 (3.6%); *p* = 0.169], allergic rhinitis [2 (8.3%) vs. 3 (10.7%); *p* = 1.000], a history of atopic eczema [5 (20.8%) vs. 1 (3.6%); *p* = 0.080], or a family history of atopy [7 (29.2%) vs. 8 (28.6%); *p* = 1.000]. Similarly, Mann–Whitney U tests revealed no statistically significant differences in IOS z-scores. Median (IQR) values in the CAH and non-CAH AI groups, respectively, were 1.05 (1.04) and 0.66 (0.95) for R5 (*p* = 0.321), 1.18 (1.27) and 0.72 (1.36) for R20 (*p* = 0.581), 1.15 (0.94) and 0.69 (1.47) for R5–R20 (*p* = 0.413), −1.30 (0.72) and −1.01 (1.05) for X5 (*p* = 0.078), −0.86 (1.00) and −0.97 (1.32) for X20 (*p* = 0.192), 1.37 (0.45) and 1.20 (0.60) for Fres (*p* = 0.316), and 1.00 (0.90) and 0.88 (1.02) for AX (*p* = 0.250) (Table 3).

In a sensitivity analysis excluding the five participants with asthma (n = 47), the frequency of IOS measurements outside the corresponding reference limits remained similar to that observed in the full cohort (R5, 19.1%; R20, 12.8%; R5–R20, 19.1%; X5, 19.1%; X20, 14.9%; AX, 17.0%; and Fres, 8.5%). Comparisons between the CAH and non-CAH AI groups also remained nonsignificant for all IOS z-scores (all *p* > 0.1), indicating that the principal findings were not materially influenced by the inclusion of participants with asthma.

## 4. Discussion

In this study, airway mechanics were assessed using impulse oscillometry in a heterogeneous group of children with adrenal insufficiency, and a subset of participants had IOS measurements falling outside published parameter-specific reference limits. A notable descriptive finding was that approximately one in five patients had R5, R5–R20, or X5 values outside the limits defined by the corresponding parameter-specific reference equations. In contrast, no significant difference was found in IOS z-scores between the congenital adrenal hyperplasia (CAH) and non-CAH AI groups.

The effects of glucocorticoids on lung development and airway physiology have been known for many years. During the fetal period, cortisol is one of the key regulators of surfactant synthesis, alveolar maturation, and type II pneumocyte differentiation. However, it has been demonstrated that glucocorticoids also play an important role in the postnatal period in maintaining airway epithelial integrity, suppressing the inflammatory response, remodeling the extracellular matrix, and regulating bronchial smooth muscle tone [1,2,3,19]. It is therefore thought that chronic cortisol deficiency may have long-term effects not only on the metabolic and cardiovascular systems but also on the respiratory system. However, studies evaluating pulmonary function in children with AI are quite limited, and the vast majority of existing studies have focused solely on conventional spirometry.

In our study, X5 was the parameter most frequently falling outside the applied reference limits, followed by R5 and R5–R20. IOS parameters reflecting peripheral airway mechanics showed the highest frequency of measurements outside the applied reference limits. Similar oscillometric findings have been reported in several pediatric conditions, including asthma, obesity, bronchopulmonary dysplasia, and cystic fibrosis [8,9,10,20]. However, these observations should be interpreted as descriptive. For each parameter assessed using a one-sided 95% reference limit, approximately 5% of healthy individuals matching the reference distribution would be expected to fall outside that limit. Assessing multiple correlated parameters also increases the probability of identifying at least one measurement outside the reference limits. Without a concurrently recruited healthy control group, our findings cannot establish an increased prevalence of peripheral airway dysfunction in children and adolescents with AI or preferential involvement of the peripheral airways.

The mechanisms underlying the observed oscillometric findings remain unclear. Experimental studies suggest that cortisol deficiency may influence inflammatory cytokine production, airway epithelial repair, extracellular matrix metabolism, adrenergic receptor expression, and bronchial smooth muscle function [5,6]. However, these mechanisms remain speculative in this cohort, in which all participants were receiving hydrocortisone replacement therapy. Exploratory analyses detected no statistically significant correlations between current hydrocortisone dose and absolute X5, R5–R20, or AX values. These unadjusted analyses, together with the absence of data on disease duration, cumulative glucocorticoid exposure, and hormonal control, prevent differentiation of the contributions of the underlying disease, treatment, and other factors.

No statistically significant differences in IOS z-scores were detected between the CAH and non-CAH AI groups, although the small subgroup sizes (n = 24 and n = 28, respectively) limit the ability to detect potentially clinically relevant differences. However, the non-CAH AI group comprised distinct disorders, including panhypopituitarism, craniopharyngioma, and adrenoleukodystrophy, with different pathophysiological mechanisms, comorbidity profiles, and concomitant treatments. This heterogeneity may obscure disease-specific associations and limit interpretation of the pooled comparison. Therefore, these nonsignificant findings should not be interpreted as evidence of equivalence or the absence of etiological differences in respiratory mechanics.

The clinical significance of the observed IOS findings remains uncertain. Although associations between peripheral airway dysfunction and respiratory symptoms, exercise intolerance, or reduced quality of life have been reported in other populations [10,21,22], these relationships and their prognostic implications were not assessed in our cohort. Measurements outside the applied reference limits alone do not establish clinically silent airway dysfunction or support routine IOS screening in children and adolescents with AI. Nevertheless, IOS requires minimal cooperation and assesses airway mechanics during tidal breathing, making it a practical research tool, particularly in younger children. Prospective controlled studies incorporating conventional pulmonary function testing and longitudinal respiratory outcomes are needed to determine the clinical relevance of these findings.

This study has several strengths. Firstly, the systematic evaluation of IOS parameters in a heterogeneous pediatric population encompassing different etiologies of AI fills an important gap in the literature. Furthermore, all IOS measurements were performed according to standardized technical quality criteria, and the use of published parameter-specific reference equations provided a structured framework for interpreting the oscillometric findings. In addition, the separate analysis of the CAH and non-CAH AI groups is one of the study’s original features.

This study has several limitations. Its cross-sectional design precludes causal inference and assessment of longitudinal respiratory outcomes. The small sample size and etiological heterogeneity limited statistical power, precision, and condition-specific analyses. The absence of concurrently recruited healthy controls prevents determination of whether measurements outside reference limits are more frequent or pronounced in participants with AI than in healthy peers. Although subgroup comparisons used IOS z-scores, the imbalance in sex distribution between the CAH and non-CAH AI groups warrants consideration, as the applied reference equations may not fully eliminate residual confounding. Without concurrent spirometry, we could not establish whether oscillometric findings accompanied airflow obstruction or occurred despite normal spirometry; therefore, these results cannot demonstrate that IOS detects abnormalities missed by spirometry or has greater sensitivity for peripheral airway dysfunction. Other complementary respiratory assessments were unavailable, and associations with inflammatory biomarkers, hormonal control, and disease duration could not be examined. Reference-related uncertainty also warrants consideration. Turkish population-specific IOS equations covering the cohort’s entire age range were unavailable; therefore, software-implemented age-specific reference sets from non-Turkish populations were used, while R5–R20 and AX were evaluated using the Gochicoa-Rangel equations. Differences in reference populations, devices, protocols, and statistical models may influence predicted values and classifications, and identical z-score thresholds do not make these reference sets interchangeable. Furthermore, R5–R20 and AX z-scores were generated using an external online calculator without independent verification against the published equations, limiting computational reproducibility. Accordingly, abnormality frequencies across parameters should be compared cautiously and should not be interpreted as population-specific estimates of peripheral airway dysfunction in Turkish children with AI. Finally, multiple comparisons were performed without multiplicity adjustment; nominal *p*-values are exploratory and do not provide confirmatory evidence.

## 5. Conclusions

This exploratory study describes IOS findings in a heterogeneous cohort of children with adrenal insufficiency. Measurements outside selected parameter-specific reference limits were observed in a subset of participants, but their physiological and clinical significance remains uncertain. No statistically significant differences were detected between the CAH and non-CAH AI groups; however, small subgroup sizes and potential confounding limit interpretation. These findings should be regarded as hypothesis-generating and require confirmation in prospective studies incorporating appropriately matched healthy controls, suitable reference equations, and clinically relevant respiratory outcomes.

## Figures and Tables

**Table 1:** Baseline demographic, clinical, laboratory, and treatment characteristics of children with AI (n = 52).

Variables	n (%)
Sex, male	28 (53.8)
Age, years, median [IQR, (min–max)]	16 [6, (4–18)]
Age categories	
<6 years	3 (5.8)
6–12 years	15 (28.8)
13–18 years	34 (65.4)
Height z-score, mean ± SD,	−0.53 ± 1.59
Weight z-score, mean ± SD,	−0.58 ± 2.054
Body mass index z-score, mean ± SD,	0.66 ± 1.61
Cause of adrenal insufficiency	
Congenital adrenal hyperplasia	24 (46.1)
Panhypopituitarism	5 (9.6)
Autoimmunity	2 (3.8)
Primary adrenal insufficiency	4 (7.7)
Adrenoleukodystrophy	6 (11.5)
Craniopharyngioma	6 (11.5)
Triple A syndrome	2 (3.8)
Melanocortin-2 receptor (MC2R) mutation	3 (5.7)
History of adrenal crisis	4 (7.7)
Parental consanguinity	24 (46.2)
Presence of atopy in the patient	
Asthma	5 (9.6)
Allergic rhinitis	5 (9.6)
Eczema history	6 (11.5)
Family history of atopy	15 (28.8)
Laboratory findings	
Eosinophil count, mean ± SD, cells/µL	169.2 ± 135.2
Total IgE, mean ± SD, IU/mL	111.8 ± 74.2
Treatment	
Hydrocortisone	52 (100)
Fludrocortisone	12 (23.1)
Antihypertensive	7 (13.5)
Anti-androgen	6 (11.5)
Oral Contraceptives/estrogen	6 (11.5)
Aromatase inhibitor	2 (3.8)
Testosterone	2 (3.8)
Desmopressin	6 (11.5)
Genotropin	5 (9.6)
Levothyroxine	11 (21.2)
Hydrocortisone dose, mg/m^2^/day, mean ± SD	11.51 ± 5

**Table 2:** IOS measurements and frequencies of values outside the applied reference limits in children and adolescents with adrenal insufficiency, including a sensitivity analysis excluding participants with asthma.

Variables	Impulse Oscillometry Parameters	Abnormality Criterion (ULN/LLN z-Score)	Values Outside Reference Limits	
			Full Cohort (n = 52) n (%)	Excluding Asthma (n = 47) n (%)
R5 (kPa·L^−1^ s), median (IQR)	0.49 (0.32)	z > +1.645	10 (19.2)	9 (19.1)
R20 (kPa·L^−1^ s), median (IQR)	0.29 (0.13)	z > +1.645	7 (13.5)	6 (12.8)
R5–R20 (kPa·L^−1^ s), median (IQR)	0.20 (0.16)	z > +1.645	10 (19.2)	9 (19.1)
X5 (kPa·L^−1^ s), median (IQR)	−0.13 (0.11)	z < −1.645	11 (21.2)	9 (19.1)
X20 (kPa·L^−1^ s), median (IQR)	−0.01 (0.08)	z < −1.645	8 (15.4)	7 (14.9)
AX (kPa·L^−1^), median (IQR)	1.74 (1.30)	z > +1.645	8 (15.4)	8 (17.0)
Fres (Hz), median (IQR)	20.8 (5.32)	z > +1.645	4 (7.7)	4 (8.5)
Participants with abnormal IOS parameters				
≥1 abnormal parameter			18 (34.6)	16 (34.0)
≥2 abnormal parameters			8 (15.4)	7 (14.9)
≥3 abnormal parameters			7 (13.4)	6 (12.8)

Values are presented as median (interquartile range (IQR)) or n (%). Abnormal results indicate measurements outside the applied parameter-specific reference limits and do not independently establish respiratory disease.

**Table 3:** Comparison of demographic, clinical, and impulse oscillometry characteristics between children with congenital adrenal hyperplasia and non-CAH adrenal insufficiency.

Variables	Congenital Adrenal Hyperplasia (n = 24)	Non-CAH Adrenal Insufficiency (n = 28)	*p*-Value
Male sex, n (%)	9 (37.5)	19 (67.9)	0.056
Age, years, median (IQR)	14.5 (6)	16 (6)	0.57
Asthma, n (%)	4 (16.7)	1 (3.6)	0.169
Allergic rhinitis, n (%)	2 (8.3)	3 (10.7)	1.0
Atopic eczema history, n (%)	5 (20.8)	1 (3.6)	0.08
Family history of atopy, n (%)	7 (29.2)	8 (28.6)	1.0
R5 z-score, median (IQR)	1.05 (1.04)	0.66 (0.95)	0.321
R20 z-score, median (IQR)	1.18 (1.27)	0.72 (1.36)	0.581
R5–R20 z-score, median (IQR)	1.15 (0.94)	0.69 (1.47)	0.413
X5 z-score, median (IQR)	−1.30 (0.72)	−1.01 (1.05)	0.078
X20 z-score, median (IQR)	−0.86 (1.0)	−0.97 (1.32)	0.192
AX z-score, median (IQR)	1.0 (0.90)	0.88 (1.02)	0.250
Fres z-score, median (IQR)	1.37 (0.45)	1.20 (0.60)	0.316

## Data Availability

The data supporting the findings of this study are available from the corresponding author upon reasonable request, subject to applicable ethical and institutional restrictions. The data are not publicly available because they contain sensitive information about pediatric participants.

## Supplementary Materials

The following supporting information can be downloaded at: https://www.mdpi.com/article/10.3390/life16101764/s1, Supplementary Materials S1: illustrative example of the calculator inputs and outputs.
