# Supplementary material for: Impulse Oscillometry Findings in Children and Adolescents with Adrenal Insufficiency: An Exploratory Cross-Sectional Study
Source: Life (Basel). 2026 Oct 21;16(10):1764. doi: 10.3390/life16101764 (PMC13706464; doi:10.3390/life16101764)
Supplement: Supplementary file 1 [file life-16-01764-s001.zip › life-4578118-supplementary.pdf]

IOS Z-Score Raporu

5 Ekim 2026

CINSIYET	YAŞ	BOY	VKİ
Kadın	11 yıl	144 cm	18.3 kg/m^2

Direnç Parametreleri

Parametre	Ölçülen	Tahmini	LLN	ULN	Z-Score	Durum
**R5**	0.5500	0.5064	0.3353	0.6774	+0.42	Normal
**R20**	0.3600	0.3754	0.2463	0.5046	-0.20	Normal
**R5-R20**	0.1900	0.1310	0.0095	0.2526	+0.80	Normal

Reaktans Parametreleri

Parametre	Ölçülen	Tahmini	LLN	ULN	Z-Score	Durum
**X5**	-0.2100	-0.1776	-0.2722	-0.0831	-0.56	Normal
**X20**	-0.0800	0.0131	-0.0446	0.0708	-2.65	Anormal
**Fres**	26.9000	18.5878	13.3241	23.8514	+2.60	Anormal
**AX**	2.4600	0.8763	0.4105	1.8706	+2.24	Anormal

Bu rapor yalnızca bilgilendirme amaçlıdır ve tıbbi tavsiye niteliği taşımaz. Klinik kararlar için uzman hekime danışınız.

IOS Z-Score Calculator — Gochicoa-Rangel et al. ERJ Open Res 2023; 9: 00503-2023
Geliştirici: Uzm.Dr.Cihangir Şahin

IOS Z-Score
Calculator

Sorumluluk Reddi | TR | EN

IOS Z-Score Hesaplayıcı

İmpuls Osilometri referans denklemlerine dayalı Z-score, tahmini değer, LLN ve ULN hesaplama aracı

2.7 – 90 yaş | 7 parametre

Hesaplama Sonuçları

Nota Kopyala | PDF Çıktı

Hesaplama | Sonuçlar 7 | Bronkodilatör | Toplu Hesaplama | Referans

Parametre	Ölçülen	Tahmini	LLN	ULN	Z-Score	Durum
R20	0.360	0.375	0.248	0.505	-0.20	Normal
R5-R20	0.190	0.131	0.009	0.253	+0.80	Normal
X5	-0.210	-0.178	-0.272	-0.083	-0.56	Normal
X20	-0.080	0.013	-0.045	0.071	-2.65	Anormal
Fres	26.900	18.588	13.324	23.851	+2.60	Anormal
AX	2.460	0.876	0.411	1.871	+2.24	Anormal

R5: 0.550 (Z: +0.42), R20: 0.360 (Z: -0.20), R5-R20: 0.190 (Z: +0.80), X5: -0.210 (Z: -0.56), X20: -0.080 (Z: -2.65), Fres: 26.900 (Z: +2.60), AX: 2.460 (Z: +2.24)

Direnç Parametreleri

R5 — Normal
kPa/(L·s^{-1})
Z-Score: +0.42

R20 — Normal
kPa/(L·s^{-1})
Z-Score: -0.20

-4 0 +4

Ölçülen:	0.5500	Tahmini:	0.5064
LLN:	0.3353	ULN:	0.6774
% Tahmini:	108.6%	Yaş Aralığı:	7.37-19.1

-4 0 +4

Ölçülen:	0.3600	Tahmini:	0.3754
LLN:	0.2463	ULN:	0.5046
% Tahmini:	95.9%	Yaş Aralığı:	7.66-17.85

R5-R20

— Normal

kPa/(L·s^{-1})

Z-Score: +0.80

-4 0 +4

Ölçülen:	0.1900	Tahmini:	0.1310
LLN:	0.0095	ULN:	0.2526
% Tahmini:	145.0%	Yaş Aralığı:	2.7-23.55

Reaktans Parametreleri

X5

— Normal

kPa/(L·s^{-1})

Z-Score: -0.56

-4 0 +4

Ölçülen:	-0.2100	Tahmini:	-0.1776
LLN:	-0.2722	ULN:	-0.0831
% Tahmini:	118.2%	Yaş Aralığı:	5.3-18.68

X20

Anormal

kPa/(L·s^{-1})

Z-Score: -2.65

-4 0 +4

Ölçülen:	-0.0800	Tahmini:	0.0131
LLN:	-0.0446	ULN:	0.0708
% Tahmini:	-611.2%	Yaş Aralığı:	5.3-20.99

Fres

Anormal

Hz

Z-Score: +2.60

-4 0 +4

Ölçülen:	26.9000	Tahmini:	18.5878
LLN:	13.3241	ULN:	23.8514
% Tahmini:	144.7%	Yaş Aralığı:	9.7-20.98

AX

Anormal

kPa/L

Z-Score: +2.24

-4 0 +4

Ölçülen:	2.4600	Tahmini:	0.8763
LLN:	0.4105	ULN:	1.8706
% Tahmini:	280.7%	Yaş Aralığı:	6.99-23.21
